# HIV epidemics in Shenzhen and Chongqing, China

**DOI:** 10.1371/journal.pone.0192849

**Published:** 2018-02-15

**Authors:** Shu Yang, Alice P. Y. Chiu, Qianying Lin, Ziqian Zeng, Yafei Li, Yao Zhang, Zhengrong Yang, Lin Yang, Daihai He

**Affiliations:** 1 Chengdu Medical College, Chengdu, Sichuan, China; 2 Department of Applied Mathematics, Hong Kong Polytechnic University, Hung Hom, Kowloon, Hong Kong (SAR) China; 3 Department of Epidemiology, College of Preventive Medicine, Third Military Medical University, Chongqing 400038, China; 4 Center for Disease Control and Prevention, Shenzhen, China; 5 School of Nursing, Hong Kong Polytechnic University, Hong Kong (SAR) China; Georgia State University, UNITED STATES

## Abstract

**Objective:**

Men who have sex with men (MSM) and heterosexuals are the populations with the fastest growing HIV infection rates in China. We characterize the epidemic growth and age patterns between these two routes from 2004 to 2015 in Chongqing and Shenzhen, China.

**Design and methods:**

Data were downloaded from the National HIV/ AIDS Comprehensive Response Information Management System. For the new HIV diagnoses of heterosexuals and MSM in both cities, we estimated the growth rates by fitting different sub-exponential models. Heat maps are used to show their age patterns. We used histograms to compare these patterns by birth cohort.

**Results:**

The MSM epidemics grew significantly in both cities. Chongqing experienced quadratic growth in HIV reported cases with an estimated growth rate of 0.086 per week and a “deceleration rate” of 0.673. HIV reported cases of MSM in Shenzhen grew even more drastically with a growth rate of 0.033 per week and “deceleration rate” of 0.794. The new infections are mainly affecting the ages of 18 to 30 in Chongqing and ages of 20 to 35 in Shenzhen. They peaked in early 1990’s and mid-1990’s birth cohorts in Chongqing and Shenzhen respectively. The HIV epidemic among heterosexuals grew rapidly in both cities. The growth rates were estimated as 0.02 and 0.028 in Chongqing and Shenzhen respectively whereas the “deceleration rates” were 0.878 and 0.790 in these two places. It affected mostly aged 18 to 75 in males and 18 to 65 in females in Chongqing and aged 18 to 45 in males and 18 to 50 in females in Shenzhen in 2015. In Chongqing, the heterosexual female epidemics display two peaks in HIV diagnoses in the birth cohorts of early 1950’s and early 1980’s, with heterosexual male epidemics peaked in early 1940’s and early 1960’s. The heterosexual male and female epidemics display higher rates in the birth cohort 1940-1960, than the birth cohort 1960-1990. It peaked in birth cohorts of 1950’s and 1980’s in Shenzhen.

**Conclusions:**

We revealed striking differences in epidemic growth and age patterns of the HIV epidemics in these two cities. Our results may be used to inform age-targeted public health policies to curb their epidemic growth.

## Introduction

The Human Immunodeficiency Virus (HIV) epidemic has become the greatest global public health challenge with some 2.1 million new infections in 2015 [1]. Asia and Pacific accounted for 300,000 new infections [1]. In 2015, adolescents and younger men and women aged 15 to 24 years old comprised of 14% and 20% new infections of all adults worldwide, which indicates that these two groups are at high risk of HIV infections [1]. In China, while the national HIV epidemic demonstrated a low prevalence trend, 12 of 31 provinces had reported over 10,000 cases of People living with HIV (PLHIV) or AIDS individually in 2014. The proportion of MSM cases has also risen from 2.5% to 25.8% during the same period [2].

Previous studies demonstrated significant variations in HIV prevalence trends geographically and among different routes of transmission in China. MSM prevalence has increased threefold from 2000 to 2010. In contrast, HIV prevalence amongst injection drug users (IDU) decreased outside Southwest China but remained at relatively low levels in all Chinese regions except southwest. The trend in female sex workers has stabilized at relatively low levels in all Chinese regions except Southwest [3]. Qian et al. identified six geographic epidemic clusters in China. Chongqing belongs to the same cluster as Guizhou and Sichuan. Their epidemic has shifted from IDU to sexual transmission in recent years. Shenzhen belongs to another cluster that consists of Beijing, Tianjin, Jiangsu, Zhejiang, Shanghai and Guangdong. Their epidemic has expanded from heterosexual transmission to both heterosexual and MSM transmission in the past few years [4]. Xing et al. studied the HIV epidemic among older adults and observed HIV hotspots in Chongqing, Guangxi, Henan, Yunan and Sichuan provinces [5]. Zhang et al. used spatial temporal analysis to analyse the epidemic among young people in China and found a marked increase in sexual transmission with a wide geographical variation [6].

Numerous studies have explained the HIV prevalence and trends in Chongqing and Shenzhen [7–17]. Zhang et al. investigated the age distribution of HIV cases of MSM in Chongqing, but their results were limited by the relatively small sample size and short study period [12].

Chongqing is China’s largest municipality, and one of the HIV hotspots in China. It borders Sichuan. It is located in the southwest of China. Chongqing has a total area of 82,400 km^2^. It has a population of 29,914,000, with 59.6% residing in urban areas [18]. It is one of four Chinese municipalities under the direct control of the central government. It is also the political and economic center of Southwest China [8]. Between 2007 and 2012, new HIV infections grew at an average annual rate of 19.7%, which was substantially higher than the national rate (3.13%) [15, 19, 20]. The estimated number of MSM in Chongqing is 16,767 [21]. In 2010, Chongqing attracted 1.91% of the national migrant population [22].

Shenzhen is a major city of Guangdong province in Southern China. In 1980, it was designated as China’s first special economic zone. Shenzhen was one of the world’s fastest growing cities during the 1990’s and 2000’s. It has a population of 10,778,900 [23]. Their average annual disposable income is RMB 44,652 [24]. There are an estimated 100,000 MSM in Shenzhen [25, 26]. 3.72% of the national migrant population resided in Shenzhen [22]. The city attracts MSM from different areas of China due to its relatively tolerant social atmosphere and rapid economic growth [27].

Shenzhen and Chongqing potentially represent two different paradigms in terms of HIV epidemics in China due to their different epidemic shifts and paces of economic development. In this study, we report on the epidemic growth and changing age patterns of new HIV diagnoses among heterosexuals and MSM populations in Chongqing and Shenzhen from 2004 to 2015.

## Materials and methods

### Data sources

HIV diagnosis data for Chongqing and Shenzhen were downloaded from the National HIV/AIDS Comprehensive Response Information Management System (CRIMS) of the National Centre for AIDS/STD Control and Prevention (NCAIDS), Chinese Centre for Disease Control and Prevention (CDC). The study period covered from 1 January 1995 to 31 December 2015. Variables include the date of birth of the HIV patients, date of HIV diagnosis, self-reported sexual orientation/ most probable sources of HIV transmission, gender, location of residence (i.e. Chongqing or Shenzhen). The inclusion criteria are as follows: (i) the individual resides in Chongqing or Shenzhen; and (ii) HIV was diagnosed within the study period. The data is anonymous and de-linked from individual identifiable information. In addition, we collected population demographic data, which included the age and sex distribution of these two populations from 1995 to 2014 [18, 23].

### Ethical statement

This study utilizes anonymous data and data from secondary sources which do not contain any personal identifiers. Therefore, ethical approval was not needed.

### Routes of transmission

Annual total new HIV diagnoses were compared between Chongqing and Shenzhen. We divided the number of HIV diagnosis by the corresponding annual populations in these two places [18, 23] and trends from 1995 to 2015 were explored.

### HIV epidemic growth modelling

To investigate the epidemic growth, we apply four sub-exponential models to heterosexuals and MSM in Chongqing and Shenzhen. The models are described in Chowell et al [28].

The weekly cumulative HIV cases takes the following form:

$$\begin{array}{c} y=C_{subexp}\left(t\right)={\left[r\left(1-p\right)t+A\right]}^{\frac{1}{1-p}} \end{array}$$

Thus the weekly new cases can be expressed as the first derivative of *C*_*subexp*_(*t*):

$$\begin{array}{c} c_{subexp}\left(t\right)=C_{subexp}^{\prime }\left(t\right)=r{\left[r\left(1-p\right)t+A\right]}^{\frac{p}{1-p}} \end{array}$$

where *r* denotes the growth rate, *p* denotes the tuning parameter, also known as deceleration of growth; and $A=C_{0}^{1-p}$, in which *C*_0_ is the initial case. We have *r* > 0 and 0 ≤ *p* ≤ 1. We set the model fitting period for each category to be the dates of first reported case to 31 December 2015.

Using the Levenberg-Marquardt Nonlinear Least Squares Algorithm [29], we attain the parameter estimates. This algorithm is implemented in the R statistical package “minipack.lm” (https://www.r-project.org/).

Levenberg-Marquardt algorithm was first proposed by Levenberg [29]. It is also known as the damped least square method. It is one of the most commonly used algorithm for least squares optimization, especially in non-linear curve fitting. This algorithm compromises between the Gauss-Newton algorithm and the gradient descent method. Levenberg-Marquardt algorithm could be used even when the initial parameter vector is far from the local minimum. It could still arrive at a solution, which makes it more robust than Gauss-Newton algorithm. The Levenberg-Marquardt algorithm, as in other numerical minimization algorithms, is an iterative procedure with an initial parameter vector is used to search for the minimized least squares. At each iteration, the Jacobian matrix is computed for approximating the Hessian matrix. This is similar to the Gauss-Newton algorithm. However, unlike the Gauss-Newton algorithm which implements the line search technique to search for next value, the Levenberg-Marquardt algorithm applies a modified trust region method. The sum of squares between the non-linear function and the first-order approximation is computed iteratively until the local minimum is reached.

In order to explore the complete parameter space and to locate the global minimum, we chose 50 values for both growth rate (*r*) and deceleration parameter (*p*), uniformly distributed in (0, 1). Thus, we have 2,500 initial value combinations for the Levenberg-Marquartdt algorithm. Bootstrap method is applied to obtain 95% confidence intervals (CIs) for both *r* and *p* [30]. The steps are as follows: (i) we obtain the best fit model $C_{subexp}\left(t;\hat{r},\hat{p}\right)$. (ii) Using $C_{subexp}\left(t;\hat{r},\hat{p}\right)-C_{subexp}\left(t-1;\hat{r},\hat{p}\right)$ as the Poisson mean, we can reconstruct a simulated dataset y*. (iii) Using Levenberg-Marquardt algorithm, new estimates of (r*, p*) will be computed. (iv) We repeated (ii) and (iii) for 1,000 times, then the sample 95% CI of the bootstrap estimates, (r*, p*), are used as the 95% CI for ($\hat{r}$, $\hat{p}$) of the best fit model, respectively. Similarly, we computed the 95% prediction intervals using the same generated samples y*. This is done by sampling from residuals at each data point [31].

### Age at HIV diagnosis patterns

We used heat maps to explore the changing age distribution of new HIV diagnoses from 2004 to 2015. We made comparisons between MSM, heterosexual males and heterosexual females from Chongqing and Shenzhen, respectively. In the heat maps, different colors were used to represent the number of new HIV diagnoses by age and time period. In addition, we compared the age distribution of new HIV diagnoses of these categories using histograms. To allow for better comparison between populations with different demographic structures, we applied direct age and sex standardization to compare the standardized number of HIV cases per 100,000 populations for each age. We divide the number of heterosexual HIV diagnosis in each age category by their corresponding age and gender-specific population sizes in Shenzhen and Chongqing in 2009 census [32, 33]. We carried out similar computations for the MSM HIV diagnosis using age-specific male population sizes.

## Results

**Fig 1:**
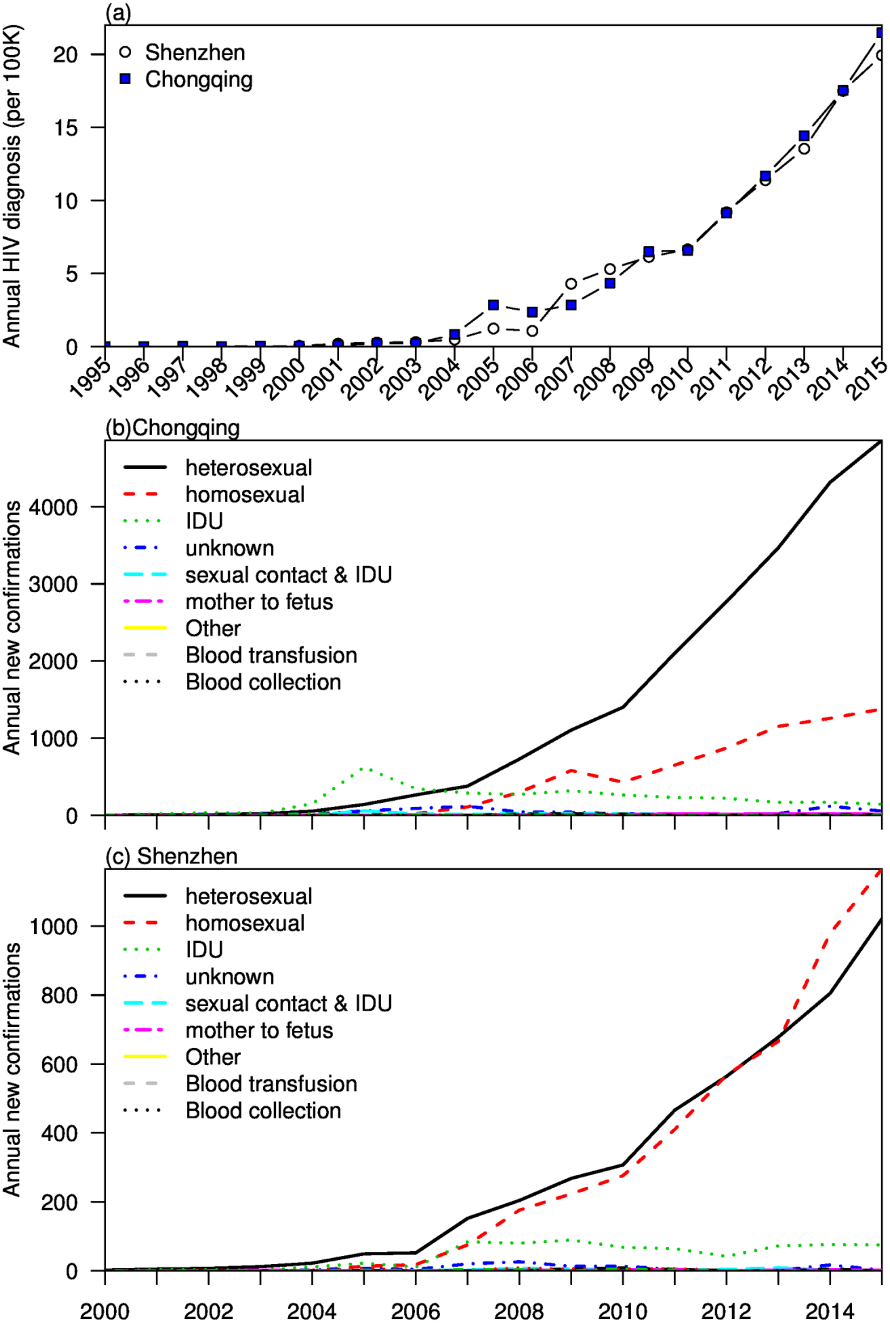
Comparison of HIV diagnosis in Chongqing and Shenzhen. (a) Annual totals after direct population standardization. (b,c) Annual confirmations by different routes of transmission.

We show the trends of new HIV diagnoses per 100,000 population in Chongqing and Shenzhen from 1995 to 2015 (Fig 1(a)). After a relatively long stagnant period from 1995 to 2003, Chongqing experienced a sharp increase in 2004 and 2005, a temporary drop in 2006, and then a period of rapid increase from 2007 to 2015. Shenzhen experienced a steady increase from 2004 to 2006, and a rapid increase from 2006 to 2015 similar to Chongqing. In Chongqing, the annual HIV diagnoses were at relatively low levels from 2000 to 2006 for all transmission routes except IDU, where there was a peak in 2005. From mid-2006 to 2015, heterosexual routes had surpassed all other routes and had risen to exceed 4,000 cases. Homosexual transmission had also risen to exceed 1,000 cases in 2015. In Shenzhen, the annual HIV diagnosis rose rapidly and homosexual transmission had surpassed heterosexual transmission since 2013. Both routes exceeded 1,000 cases in 2015 (Fig 1(b) and 1(c)).

**Fig 2:**
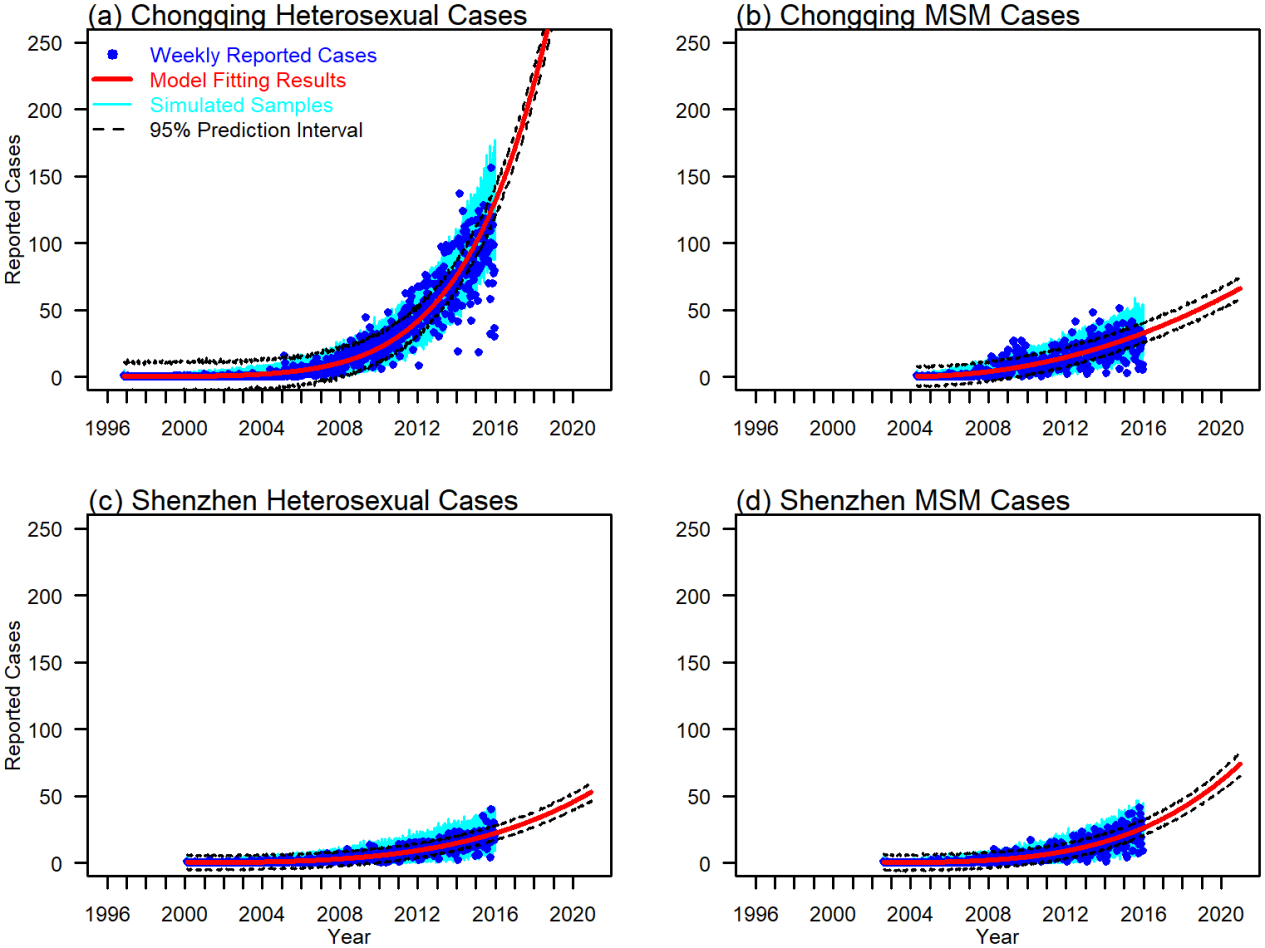
We fit the sub-exponential models to weekly reported cases of Chongqing and Shenzhen, and predict their trends from 2016 to 2020. Panel (a) and (b) present fitting results for heterosexuals and MSM populations in Chongqing, (c) and (d) present those of Shenzhen. Red solid lines show the simulation results. Blue solid dots show the weekly reported cases. Cyan lines indicate the results of 1,000 Poisson simulations. Black dashed lines represent the 95% prediction interval.

**Fig 3:**
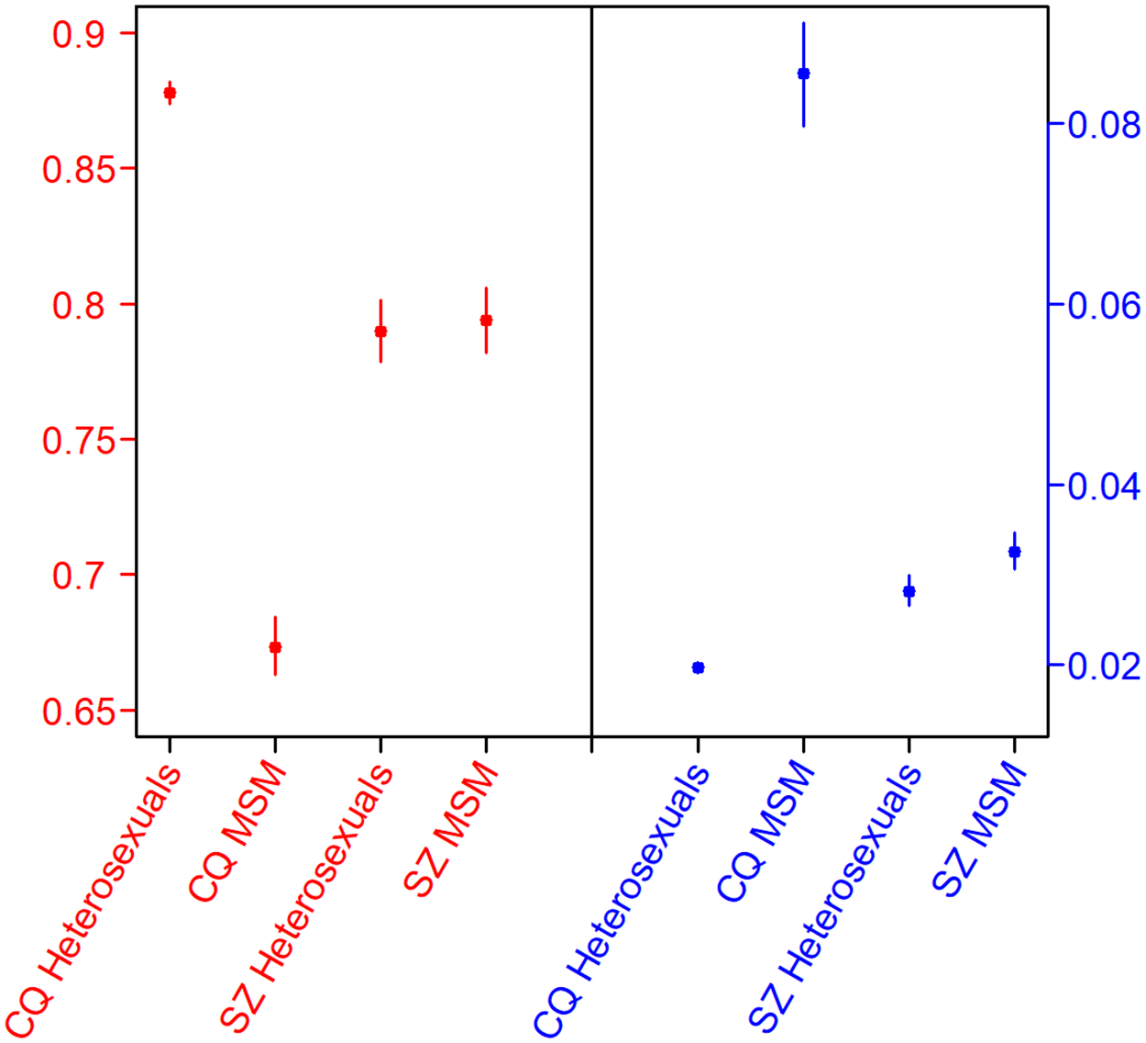
Parameter estimates and their corresponding 95% CI for each population in Chongqing(CQ) and Shenzhen(SZ).

Parameter estimates for the sub-exponential model that summarizes the epidemic growth are displayed in Table 1. Both heterosexuals and MSM in Chongqing and Shenzhen achieved deceleration (*p*) that are larger than $\frac{1}{2}$, indicating relatively fast growth. With $p=0.673\approx \frac{2}{3}$, HIV reported cases of MSM in Chongqing grew nearly quadratically and its cumulative number of reported cases fits a cubic polynomial. HIV incidence of heterosexual population in Chongqing achieved deceleration with a value close to 1, suggesting near exponential growth in early epidemic phase. We fit the reported cases to sub-exponential models and present the predicted trends from 2016 to 2020 in Fig 2. The corresponding parameter estimates and confidence intervals are shown in Fig 3. All four sub-exponential models fit well to the reported HIV cases. Also, we varied the duration of early epidemic generations and then forecasted the HIV growth trends and their 95% prediction interval (See S1 to S4 Figs).

**Table 1:** Summary of parameter estimates with 95% CIs in parentheses of the sub-exponential models for epidemic growth for heterosexuals and MSM in Chongqing and Shenzhen.

	Chongqing		Shenzhen	
	Heterosexuals	MSM	Heterosexuals	MSM
Initial Case (*A*)	1	1	1	1
Growth Rate (*r*)	0.020 (0.019, 0.020)	0.086 (0.080, 0.092)	0.028 (0.027, 0.030)	0.033 (0.031, 0.035)
Deceleration of growth (*p*)	0.878 (0.874, 0.882)	0.673 (0.662, 0.683)	0.790 (0.777, 0.802)	0.794 (0.782, 0.806)
Date of Initial Case (*s*)	14 Nov 1996	29 Apr 2004	17 Feb 2000	8 Aug 2002

**Fig 4:**
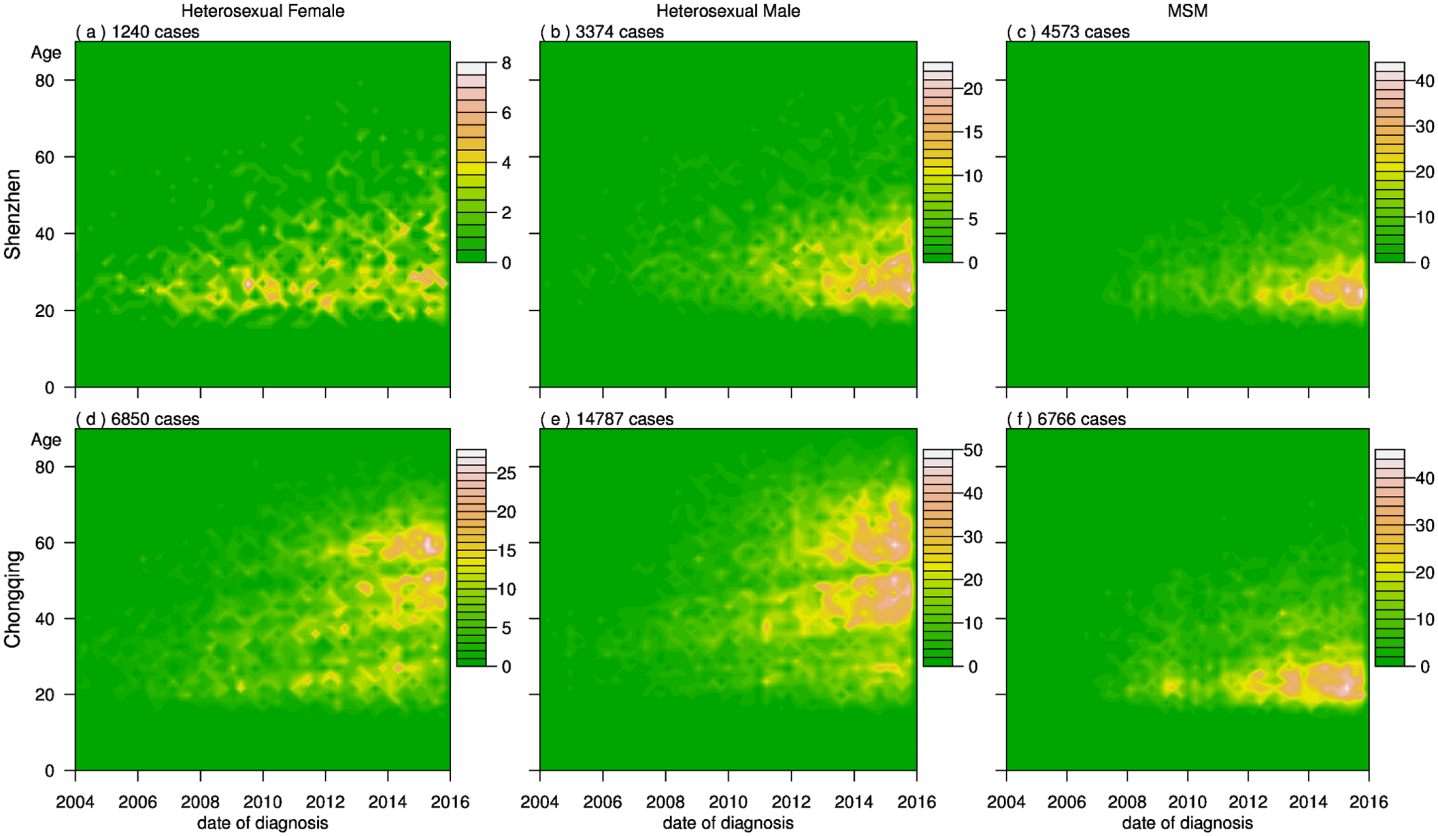
Number of cases by age at diagnosis for different routes of transmission in Chongqing and Shenzhen from their first reported cases to 2015. Within each panel, the vertical axis represents ages from 0 to 80, while the horizontal axis corresponds to the year of the analysis from 2004 to 2015. The standardized ratio of new HIV diagnoses per 100,000 population is represented by different colors, as depicted by their color codes. The heterosexual cases in Chongqing appear to be split by two strips, which is most likely due to the low birth rates during the early 1960’s and 1980’s.

The heat maps depict the changing age patterns from 2004 to 2015 (Fig 4). The HIV epidemics among heterosexuals grew rapidly in magnitude and also expanded in age range from 2004 to 2015 in both Chongqing and Shenzhen. There are distinct features in HIV epidemic patterns of heterosexual females and males in these two cities. For heterosexual females, the epidemic in Shenzhen has grown to 4-8 cases per 100,000 females per year among aged 18 to 50. In Chongqing, it has increased to 10-25 cases per 100,000 females among ages 18 to 65.

The HIV epidemic affects a wider age range and is more severe in magnitude among heterosexual males in Chongqing than those in Shenzhen. In Shenzhen, it has spread to 10-20 cases per 100,000 males per year among ages 18 to 45. In Chongqing, it has increased to 20-50 cases per 100,000 males per year among ages 18 to 75.

The HIV epidemics among MSM also grew rapidly in magnitude from 2008 to 2015, mostly concentrated in the younger populations in both cities (Fig 3(c) and 3(f)). In Shenzhen, the most severely affected were in the age range 20 to 35, with 20-40 cases per 100,000 males per year. In Chongqing, the epidemic was more concentrated for those in the age range 18 to 30, also with 20-40 cases per 100,000 males per year.

**Fig 5:**
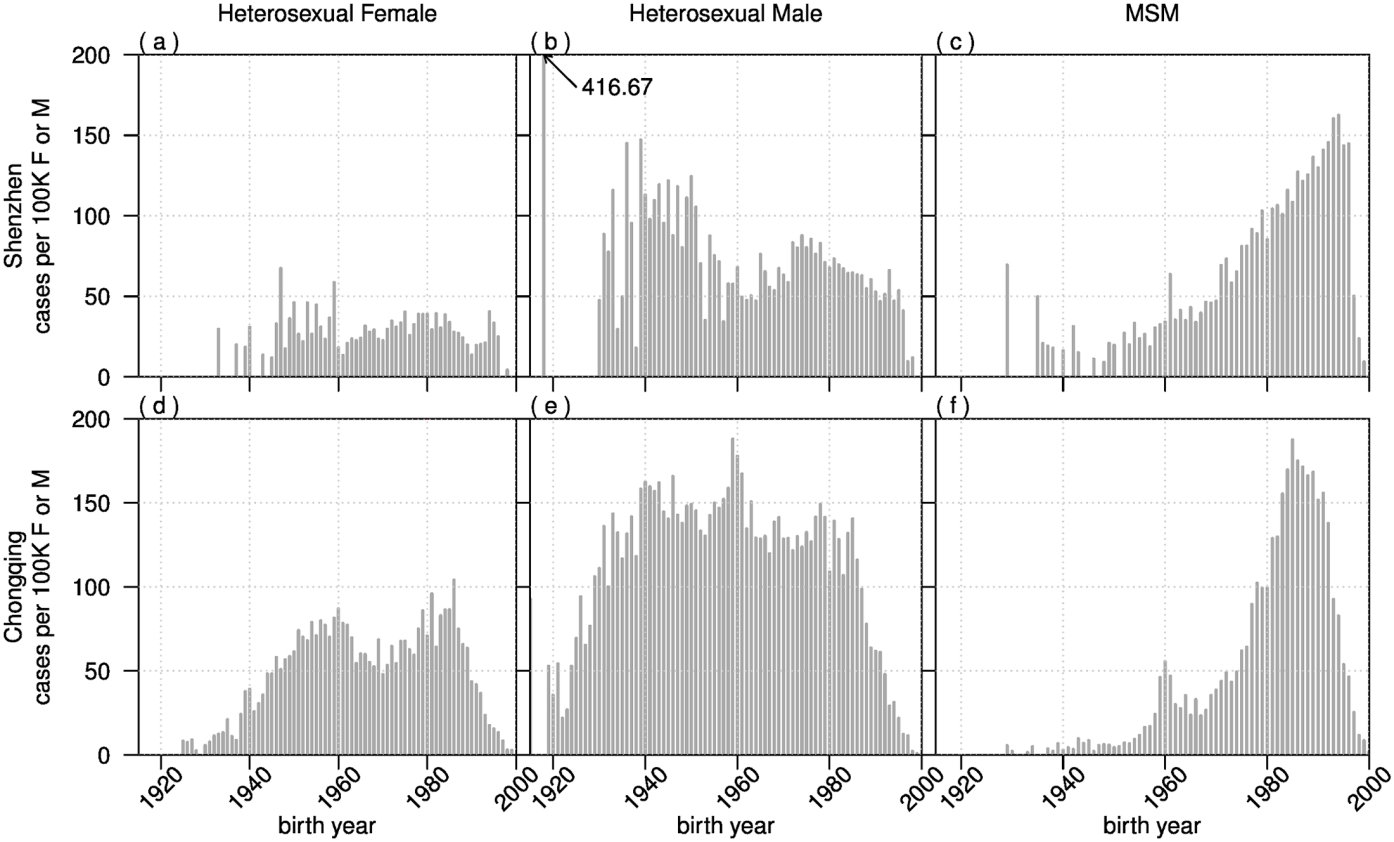
Comparison of number of cases per 100,000 males or females by age at diagnosis distribution by birth year and routes of transmission in Chongqing and Shenzhen. We used the 2010 census for the population’s age profile.

We compare the distribution of birth cohorts of the HIV new diagnoses per 100,000 males or females in Chongqing and Shenzhen (Fig 5). In Shenzhen, heterosexual females and heterosexual males display similar distribution of HIV new diagnoses, with higher rates in birth cohort 1940 to 1960, than birth cohort 1960 to 1990. Heterosexual male epidemics are also larger in magnitude than heterosexual females (Fig 5(a) and 5(b)). MSM epidemics are skewed towards the 1990’s birth cohort. There was also a high number of cases in the 1930’s birth cohort (Fig 5(c)). In Chongqing, heterosexual females epidemic display two peaks in HIV new diagnoses of 1950’s and early 1980’s (Fig 5(d), while heterosexual males epidemic peak in early 1940’s and early 1960’s (Fig 5(e)). MSM epidemics are also skewed towards the younger population of the 1990’s birth cohort (Fig 5(f)).

**Fig 6:**
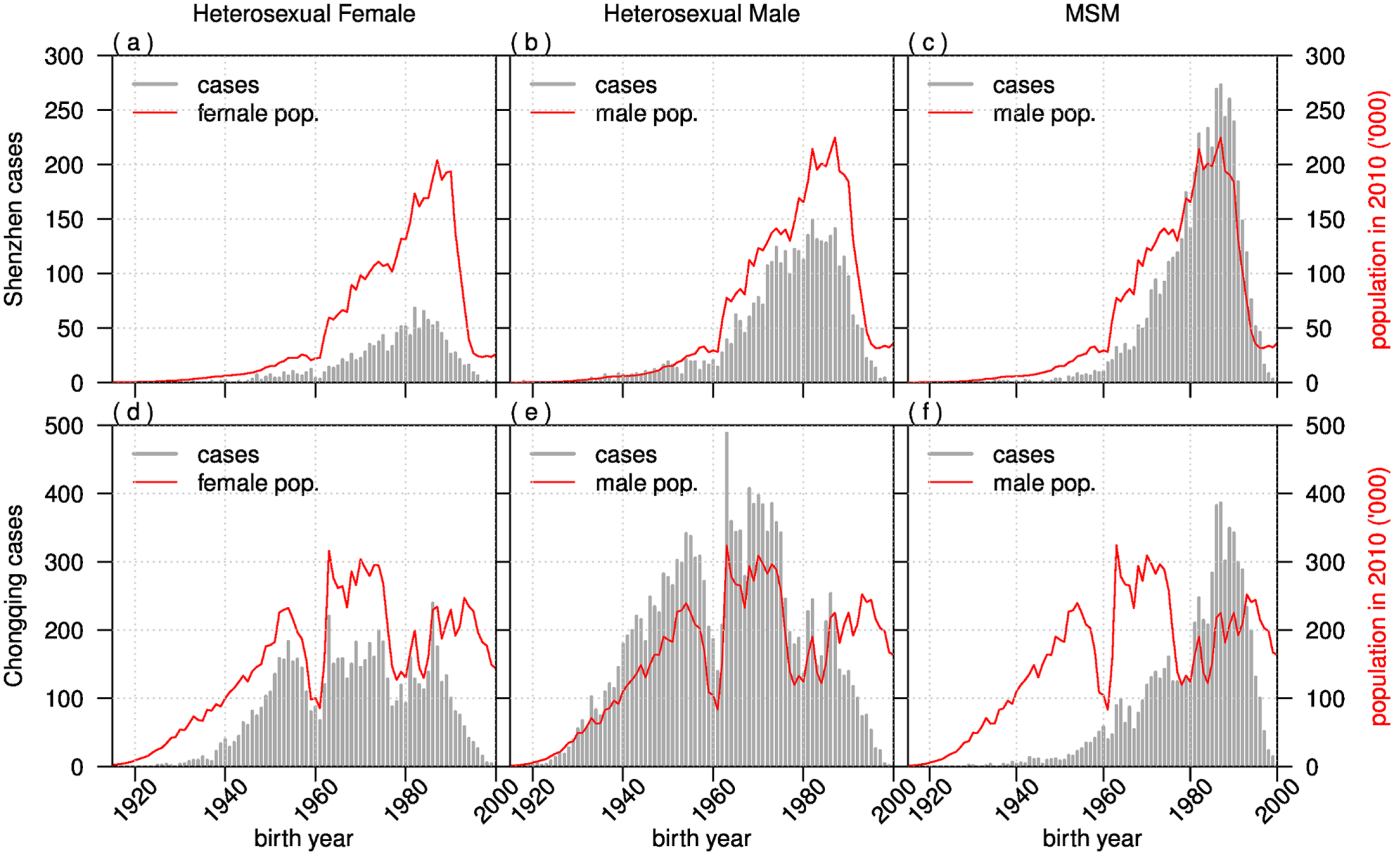
Comparison of number of cases by age at diagnosis distribution by birth year and routes of transmission in Chongqing and Shenzhen in 2010. Red curve shows the population’s age structure according to the census in 2010.

We compare the distribution of the female and male populations with the number of HIV cases in the six sub-populations in 2010 (Fig 6). In Shenzhen, male and female populations are skewed towards younger ages with a peak in the birth cohort of early 1990’s. For HIV cases, heterosexual females peaked in the birth cohort of mid-1980’s while heterosexual males displayed two peaks in early and mid-1980’s (Fig 6(a) and 6(b)). Among MSM, HIV cases peaked in the birth cohort of 1990’s (Fig 6(c)). In Chongqing, male and female populations showed two troughs in the birth cohorts of 1960’s and 1980’s, due to the famine and implementation of one-child policy, respectively. The age distributions were the highest between 1960 and 1980 birth cohort where the HIV cases also displayed a peak in the same cohort (Fig 6(d) and 6(e)). Among MSM, HIV cases also peaked in the birth cohort of 1990’s (Fig 6(f)).

## Discussion and conclusions

In this study, we examine the routes of transmission, age characteristics and epidemic growth in HIV diagnoses with data from two Chinese cities. We have shown that the HIV epidemics in Chongqing and Shenzhen have been accelerating from 2004 onwards and are dominated by sexual routes of transmission (Fig 1). We fitted sub-exponential models to both heterosexual and MSM populations in Shenzhen and Chongqing (Fig 2). The predictive powers were tested in these models (See S1 to S4 Figs). By using three to four generations of HIV reported cases as training dataset, our models were able to capture the general growth trends and arrived at accurate predictions. The discrepancies between the predicted and observed data could be due to the increased awareness of safer sex and change of HIV behavioral patterns in recent years. Colgate et al. fitted sub-exponential models to HIV incidence data in the United States in the 1980’s [34]. Viboud et al. fitted a similar model in Japan from 1985 to 2012 [35]. Both studies reported quadratic growth in cumulative HIV infections. Our results suggests that the HIV epidemics were more severe for both heterosexuals and MSM in Shenzhen and Chongqing, with their cumulative growth in HIV reported cases ranging from cubic to near exponential. By considering both growth rate and deceleration of growth, our sub-exponential models show that Chongqing heterosexuals experienced the fastest growth in HIV epidemic, which is predicted to exceed 250 weekly cases in 2020.

The heterosexual HIV epidemic is growing in magnitude and are also expanding in age range, strongly affecting the 18-45 age brackets in males and 18-50 in females in Shenzhen and 18-75 in males and 18-65 in females in Chongqing. In contrast, the MSM epidemics are much more concentrated within the younger populations, from ages 18 to 30 in Chongqing and ages 20 to 35 in Shenzhen. In 2015, HIV diagnoses in heterosexuals and MSM displayed very different birth cohort distributions: heterosexuals are more concentrated in the 1950’s and 1980’s birth cohorts in Shenzhen, and the 1960’s birth cohort in Chongqing. But for HIV epidemics in MSM, they are very skewed towards the younger population. They peak in the mid 1990’s and early 1990’s birth cohorts in Shenzhen and Chongqing, respectively.

The heterosexual HIV epidemics in these two cities displayed very different patterns. The Chongqing epidemic spread to a much wider age range, at a much larger magnitude within the older ages. These observations suggest that HIV intervention activities targeting the heterosexual populations should aim at the elderly population in Chongqing and the younger population in Shenzhen. In contrast, the MSM HIV epidemics in both cities are concentrated within the younger MSM populations, affecting slightly younger ages in Chongqing than in Shenzhen. This could be due to the relatively younger age structure in Shenzhen. These age distributions are consistent with our estimates of “deceleration of growth”. A much wider age range among heterosexuals in Chongqing results in near exponential growth in their HIV epidemics, whereas more restricted age range among MSM in Chongqing could explain their relatively slower growth. Similar age distributions contribute to moderate growth of HIV epidemics for heterosexuals and MSM in Shenzhen.

The trend towards younger age at diagnosis in both heterosexuals and MSM are also observed in the United States [36, 37]. Similarly, the median age at HIV diagnosis among MSM also declined from 38.8 years in 2007 to 35.9 years in 2009 in Victoria, Australia. This Australian study also showed that younger MSM were more likely to be never tested for HIV and to report inconsistent condom uses with their regular or casual partners [38].

The Open Door Policy in 1979 brings globalization to the Chinese economy, it also impacts on sexuality and the HIV epidemics in a number of inter-connected ways [39, 40]. Economic development leads to the financial independence of men and especially women. It brings a new way of living, such as marriage by choice and women’s control over their own reproduction. It changes sexual behavior, including earlier sexual debut [41], increases in pre-marital sex, extra-marital sex and multiple sexual partnerships [40, 42]. Homosexuality that was once obscured by the contemporary Chinese society also emerged to become a legitimate lifestyle choice [43]. However, this increase in sexual activity were not accompanied by more sex education [44, 45], which in turn fueled the HIV epidemics in both sex transmission routes.

High levels of gay mobile apps use among younger MSM could also fuel the epidemic of this sub-population. Older MSMs tend to use public toilets, parks and saunas to meet new sex partners [46]. Their sexual network is more restricted geographically. In contrast, younger MSMs often use gay apps which exposes them to a much wider and dynamic partnership network in recent years [47]. This likely explains the increasing magnitude of the younger MSM epidemics.

A major strength of our study is that we have characterized the key features, i.e. the routes, age pattern and epidemic growth, of the HIV epidemics in both cities. A major limitation is that we computed the age of HIV diagnoses rates based on the overall male population within the city, whereas if we could compute the rates from the MSM population it would provide us with a more accurate picture of the epidemiology. We also used cases of new HIV diagnoses rather than HIV incident cases. The former could vary due to HIV testing intensity and are subject to variations such as delays in diagnosis.

Our study revealed striking age patterns, and suggests targeted intervention to curb the HIV epidemic growth. Interventions need to address the age, sexual orientation of the target risk groups as well as the underlying demographic structure in both cities. In conclusion, our study provides a preliminary investigation into the HIV epidemics in Chongqing and Shenzhen which are both complex and dynamic. We gained an understanding about the changes in routes of transmission, and we examined the epidemic growth and age patterns in sexual transmission. Future work could adopt similar techniques to understand the epidemics in other HIV hotspots in other Chinese cities or provinces. This would help to guide public health policymakers towards more directed HIV preventive efforts in China.

## Supporting information

S1 Fig HIV epidemic among heterosexual population in Chongqing. We fit generalized-growth model during various epidemic phases for heterosexual reported HIV cases in Chongqing and estimated the corresponding parameters *r*, *p* and their 95% CIs. In panels (a) and (b), red solid lines indicate the parameter estimates of *r* and *p* to various epidemic generations. The shaded region and blue-dashed line indicates their corresponding 95% CIs with respect to various epidemic generations. Panels (c) to (g) present the fitting results of the generalized-growth model to epidemic generations 1 to 4, and all data, with a 95% prediction interval. Red solid lines indicate the simulation result. Blue dots indicate the weekly reported cases. Cyan lines indicate the sample of 1,000 Poisson simulations.
(PDF)

S2 Fig HIV epidemic among MSM population in Chongqing. We fit generalized-growth model during various epidemic phases for MSM reported HIV cases in Chongqing and estimated the corresponding parameters *r*, *p* and their 95% CIs. In panels (a) and (b), red solid lines indicate the parameter estimates of *r* and *p* to various epidemic generations. The shaded region and blue-dashed line indicates their corresponding 95% CIs with respect to various epidemic generations. Panels (c) to (g) present the fitting results of the generalized-growth model to epidemic generations 1 to 4, and all data, with a 95% prediction interval. Red solid lines indicate the simulation result. Blue dots indicate the weekly reported cases. Cyan lines indicate the sample of 1,000 Poisson simulations.
(PDF)

S3 Fig HIV epidemic among heterosexual population in Shenzhen. We fit generalized-growth model during various epidemic phases for heterosexual reported HIV cases in Shenzhen and estimated the corresponding parameters *r*, *p* and their 95% CIs. In panels (a) and (b), red solid lines indicate the parameter estimates of *r* and *p* to various epidemic generations. The shaded region and blue-dashed line indicates their corresponding 95% CIs with respect to various epidemic generations. Panels (c) to (g) present the fitting results of the generalized-growth model to epidemic generations 1 to 4, and all data, with a 95% prediction interval. Red solid lines indicate the simulation result. Blue dots indicate the weekly reported cases. Cyan lines indicate the sample of 1,000 Poisson simulations.
(PDF)

S4 Fig HIV epidemic among MSM population in Shenzhen. We fit generalized-growth model during various epidemic phases for MSM reported HIV cases in Shenzhen and estimated the corresponding parameters *r*, *p* and their 95% CIs. In panels (a) and (b), red solid lines indicate the parameter estimates of *r* and *p* to various epidemic generations. The shaded region and blue-dashed line indicates their corresponding 95% CIs with respect to various epidemic generations. Panels (c) to (g) present the fitting results of the generalized-growth model to epidemic generations 1 to 4, and all data, with a 95% prediction interval. Red solid lines indicate the simulation result. Blue dots indicate the weekly reported cases. Cyan lines indicate the sample of 1,000 Poisson simulations.
(PDF)
