# Supplementary figures and images for: HIV epidemics in Shenzhen and Chongqing, China
Source: PLoS One. 2018 Feb 15;13(2):e0192849. doi: 10.1371/journal.pone.0192849 (PMC5813969; doi:10.1371/journal.pone.0192849)

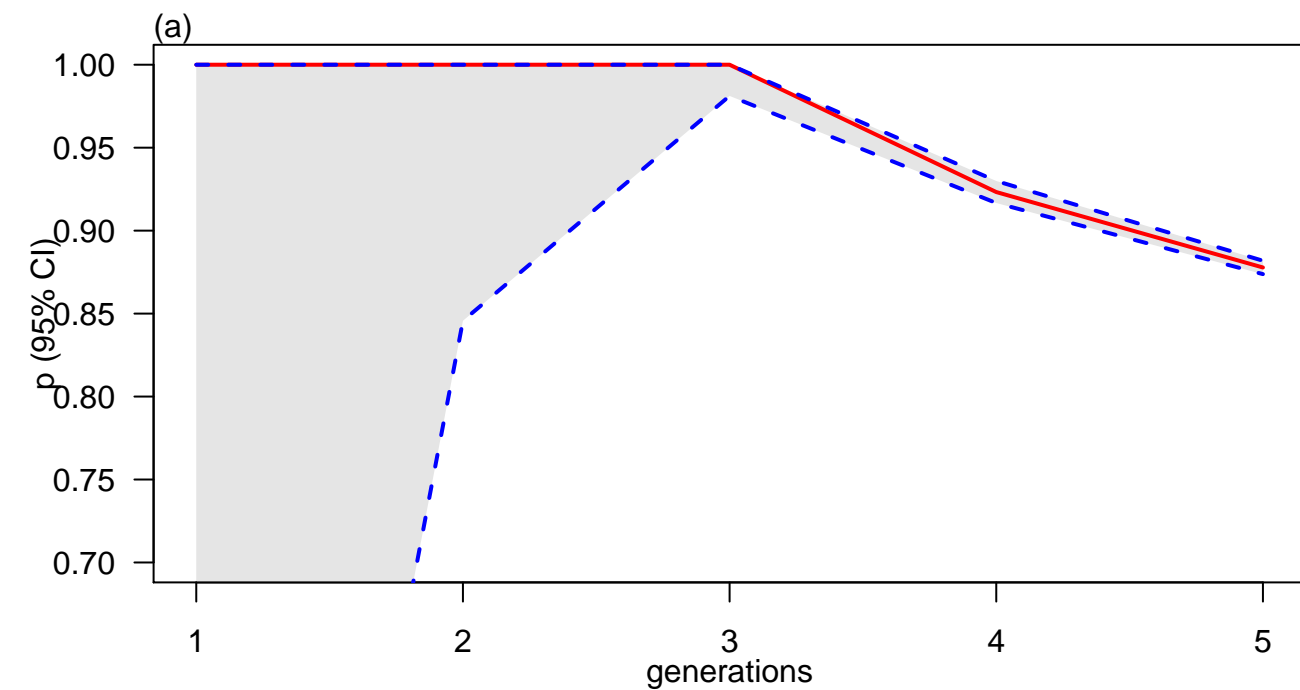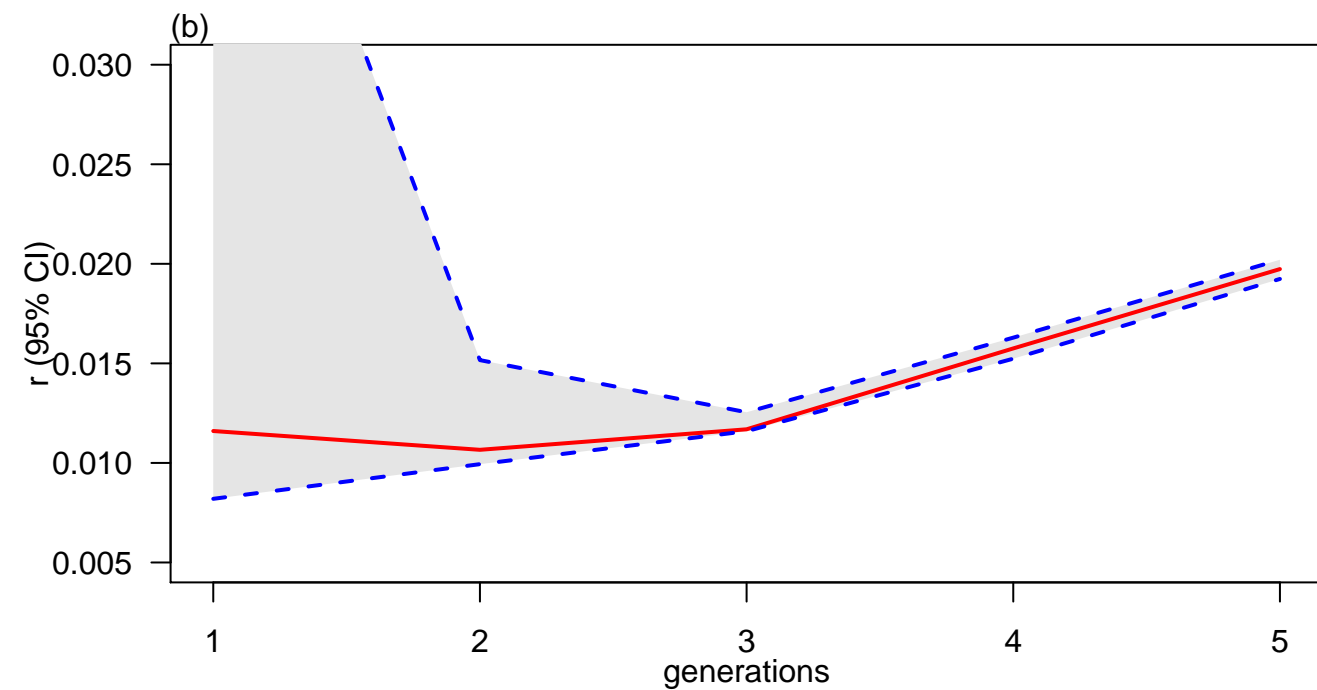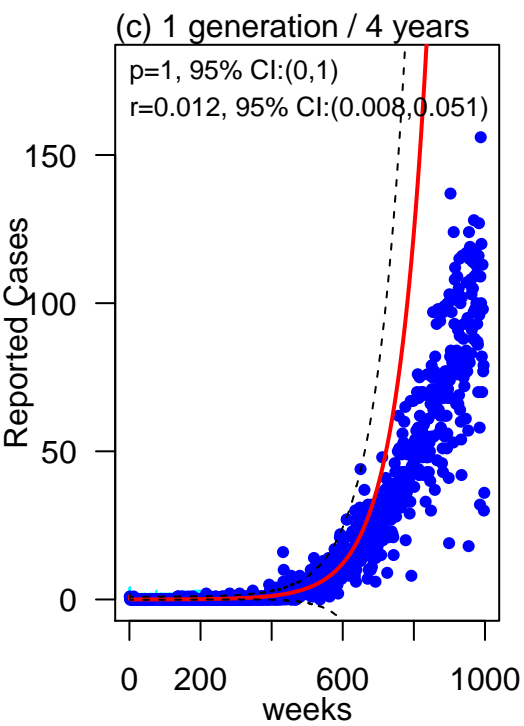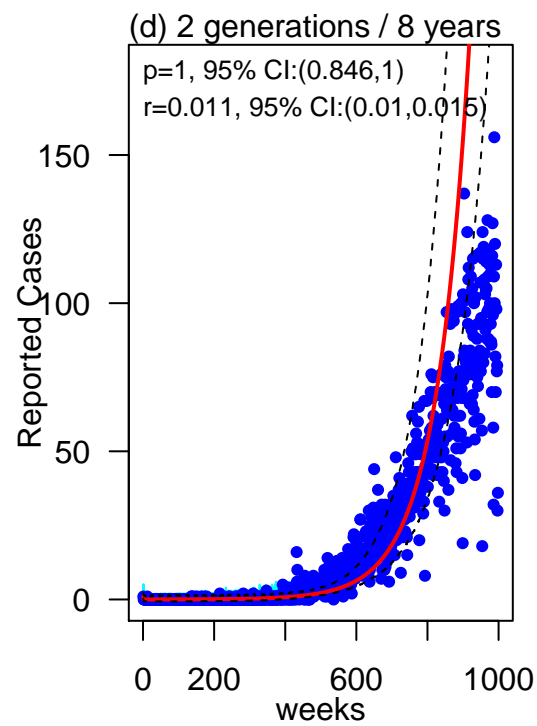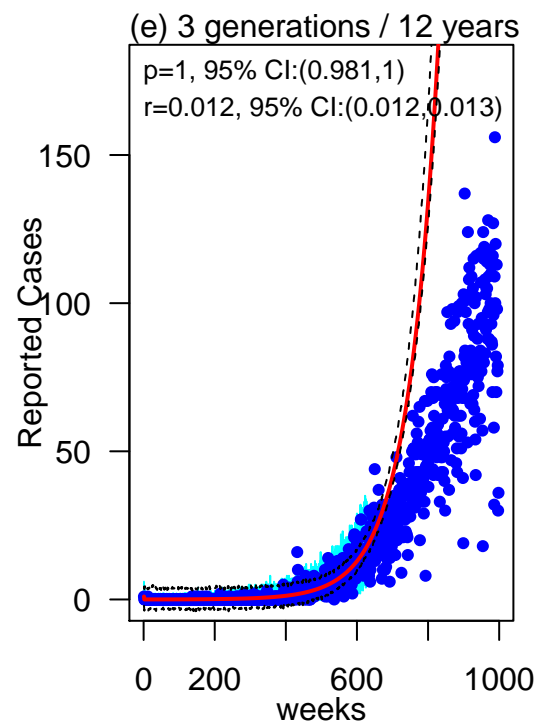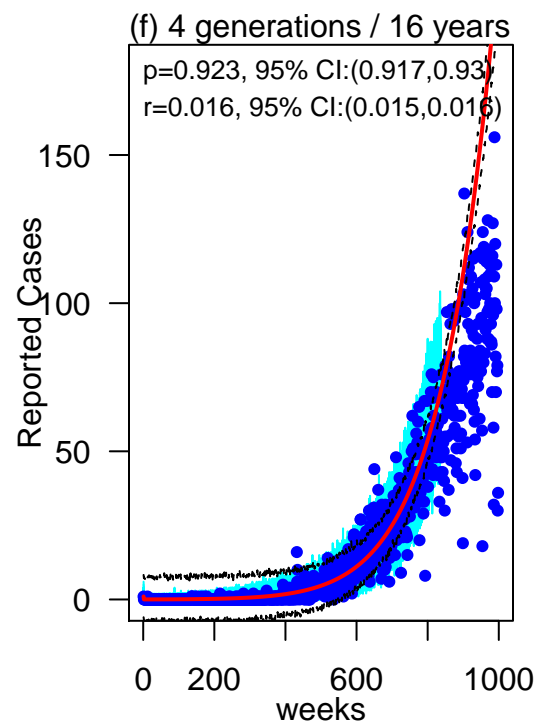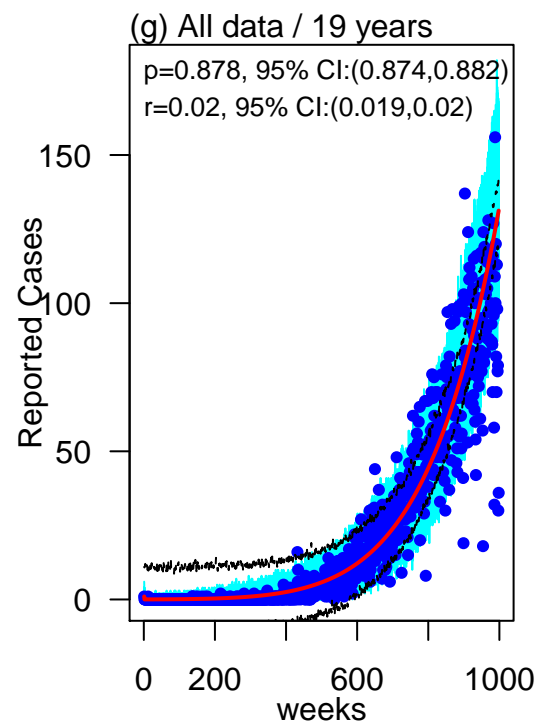

Supplement: S1 Fig — We fit generalized-growth model during various epidemic phases for heterosexual reported HIV cases in Chongqing and estimated the corresponding parameters r, p and their 95% CIs. In panels (a) and (b), red solid lines indicate the parameter estimates of r and p to various epidemic generations. The shaded region and blue-dashed line indicates their corresponding 95% CIs with respect to various epidemic generations. Panels (c) to (g) present the fitting results of the generalized-growth model to epidemic generations 1 to 4, and all data, with a 95% prediction interval. Red solid lines indicate the simulation result. Blue dots indicate the weekly reported cases. Cyan lines indicate the sample of 1,000 Poisson simulations. (PDF) [file pone.0192849.s001.pdf]

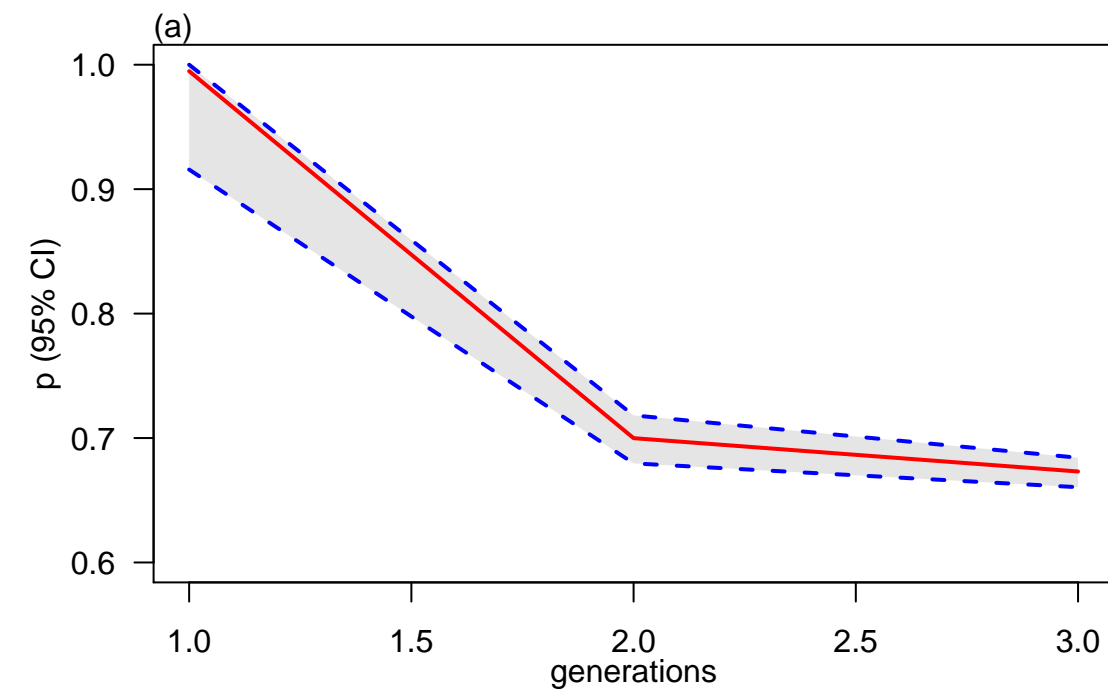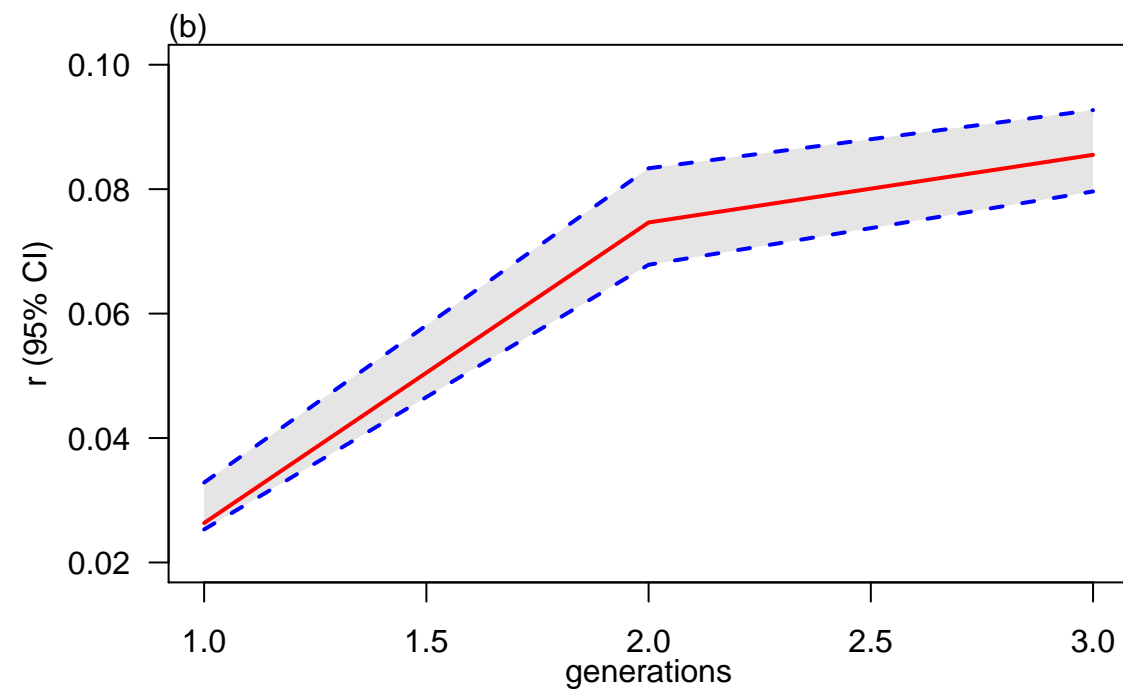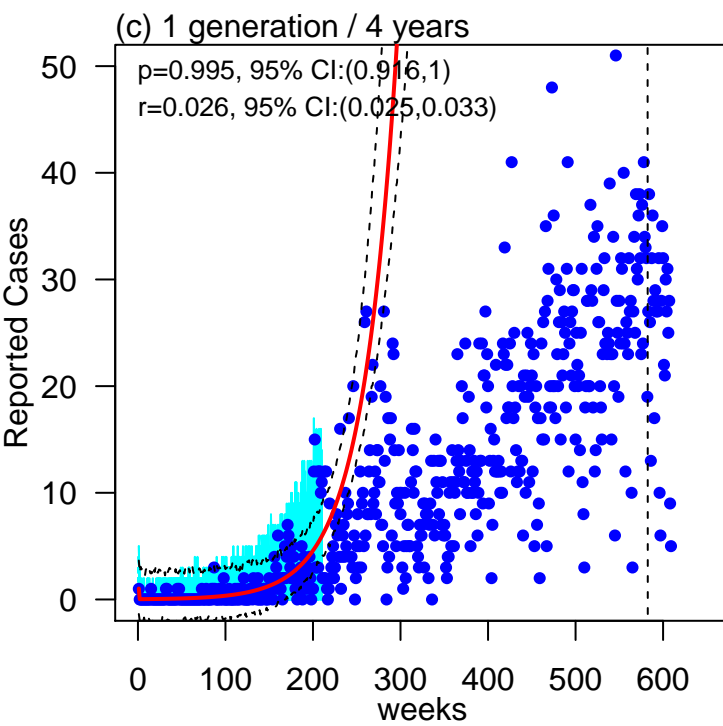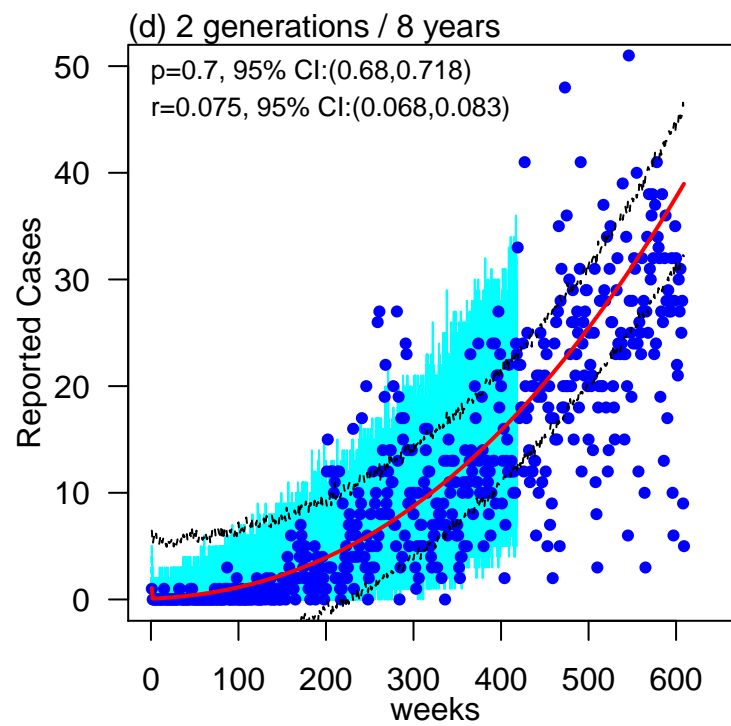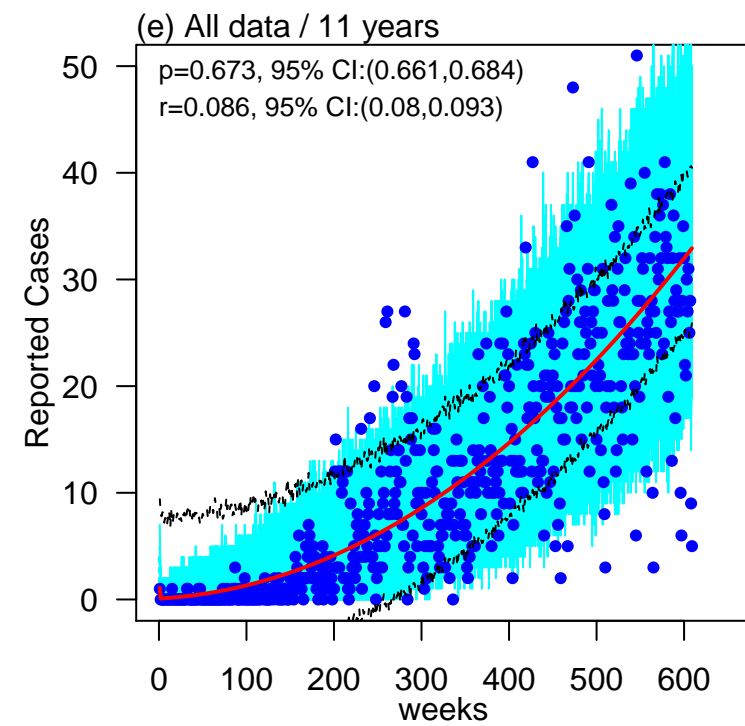

Supplement: S2 Fig — We fit generalized-growth model during various epidemic phases for MSM reported HIV cases in Chongqing and estimated the corresponding parameters r, p and their 95% CIs. In panels (a) and (b), red solid lines indicate the parameter estimates of r and p to various epidemic generations. The shaded region and blue-dashed line indicates their corresponding 95% CIs with respect to various epidemic generations. Panels (c) to (g) present the fitting results of the generalized-growth model to epidemic generations 1 to 4, and all data, with a 95% prediction interval. Red solid lines indicate the simulation result. Blue dots indicate the weekly reported cases. Cyan lines indicate the sample of 1,000 Poisson simulations. (PDF) [file pone.0192849.s002.pdf]

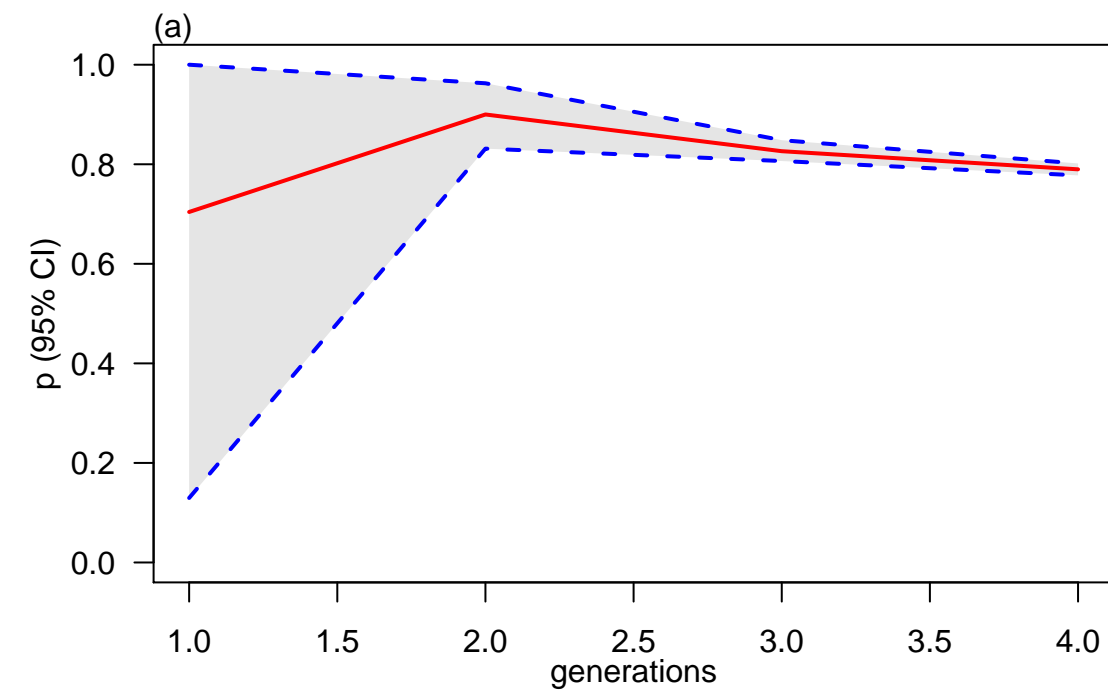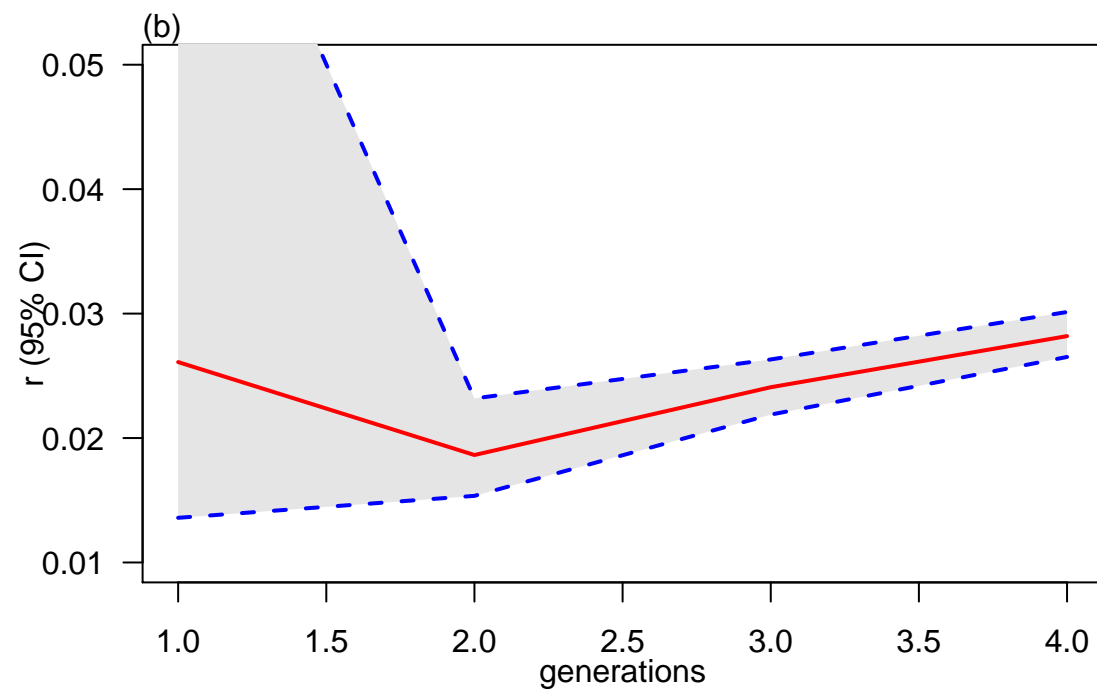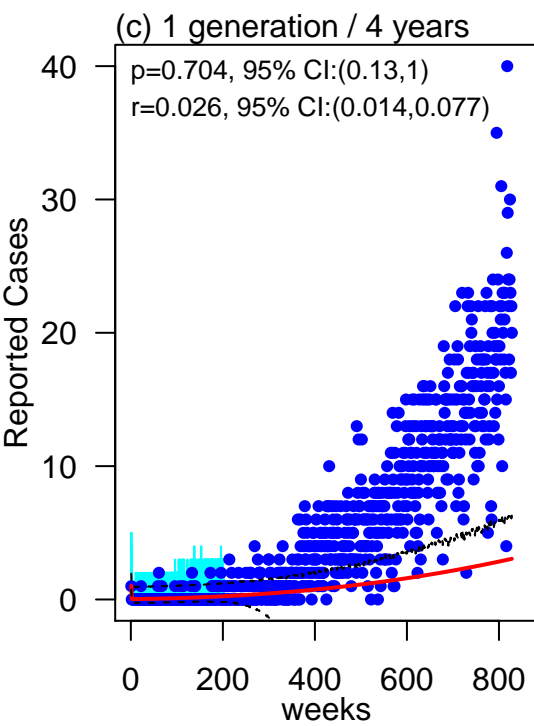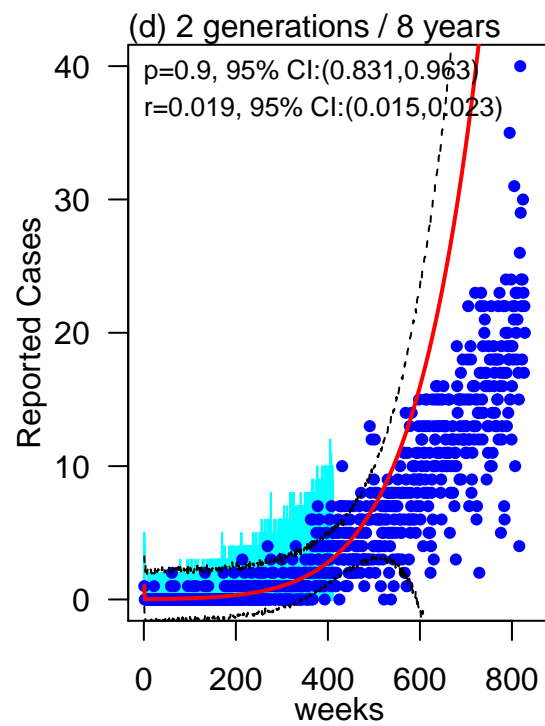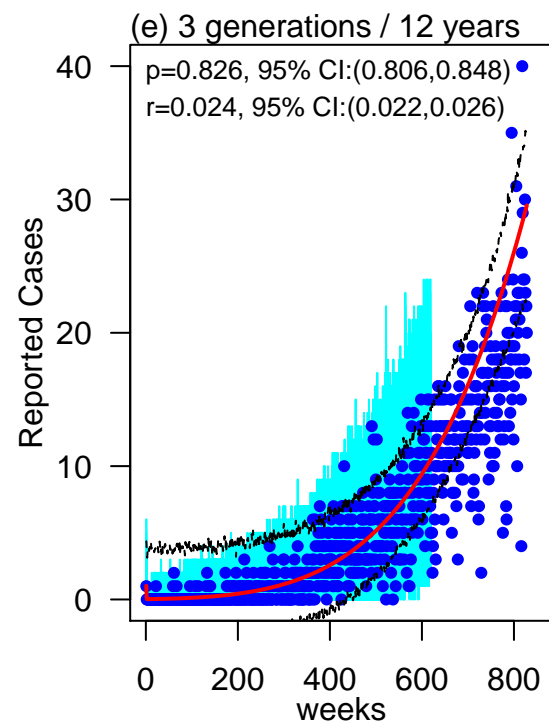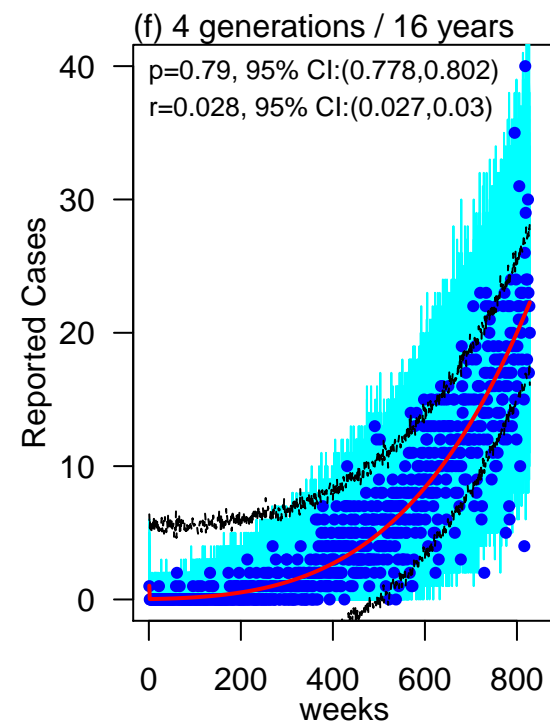

Supplement: S3 Fig — We fit generalized-growth model during various epidemic phases for heterosexual reported HIV cases in Shenzhen and estimated the corresponding parameters r, p and their 95% CIs. In panels (a) and (b), red solid lines indicate the parameter estimates of r and p to various epidemic generations. The shaded region and blue-dashed line indicates their corresponding 95% CIs with respect to various epidemic generations. Panels (c) to (g) present the fitting results of the generalized-growth model to epidemic generations 1 to 4, and all data, with a 95% prediction interval. Red solid lines indicate the simulation result. Blue dots indicate the weekly reported cases. Cyan lines indicate the sample of 1,000 Poisson simulations. (PDF) [file pone.0192849.s003.pdf]

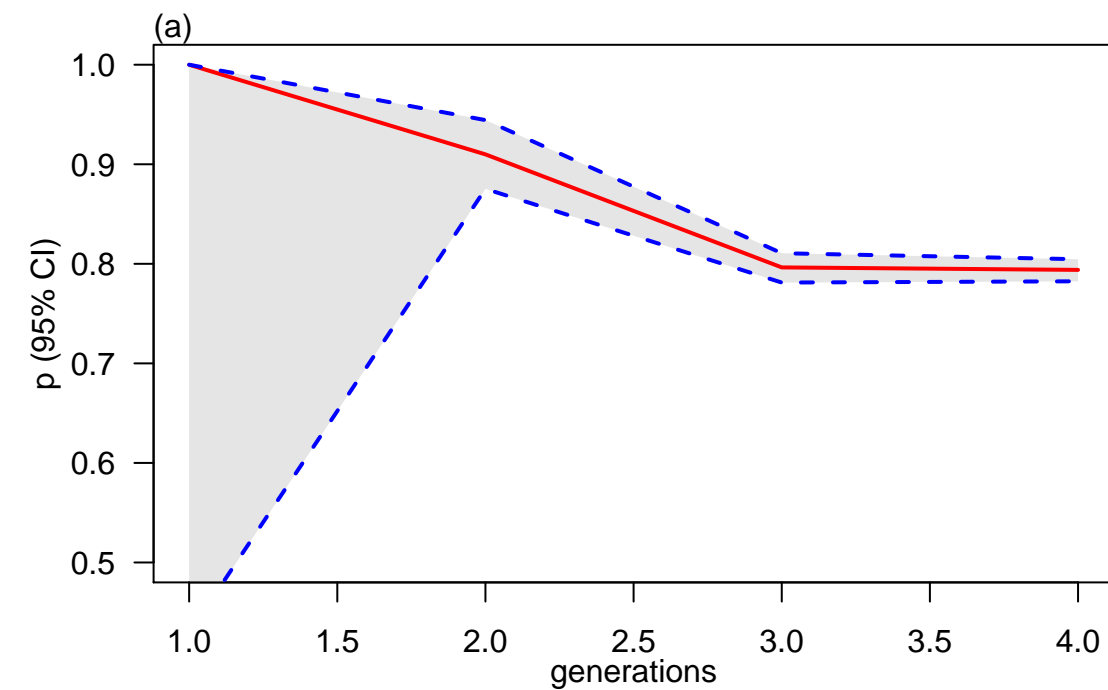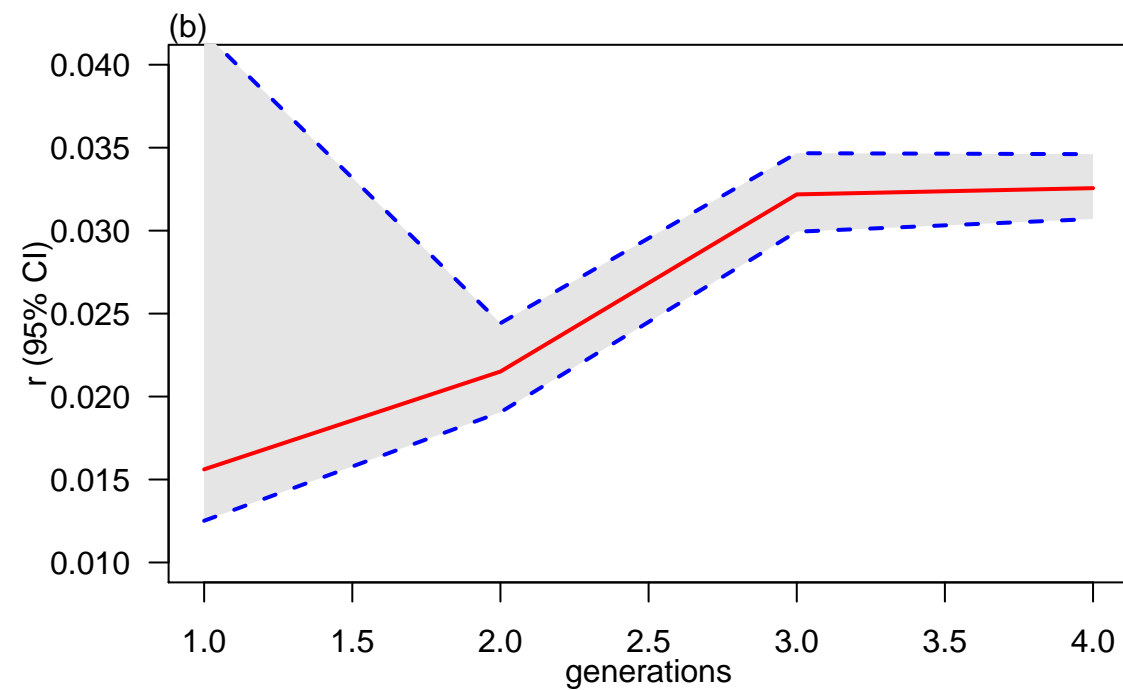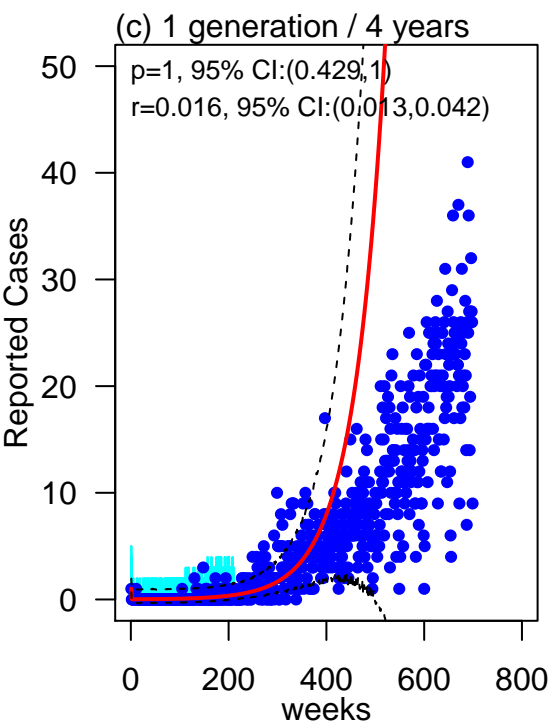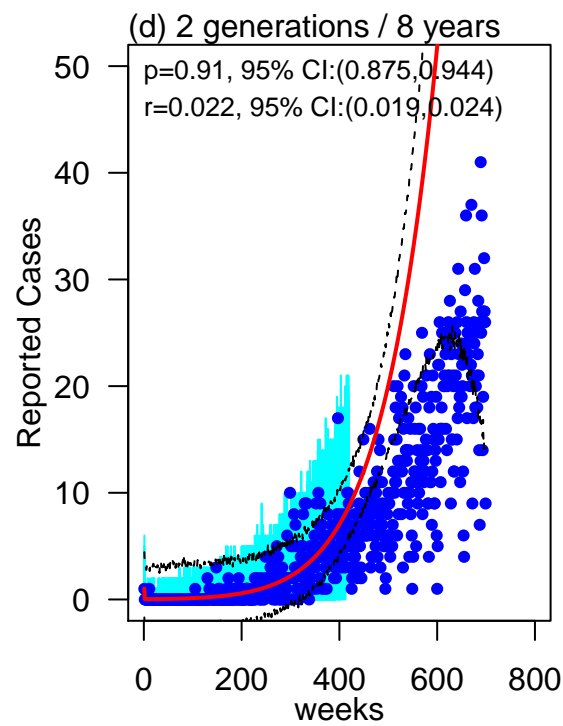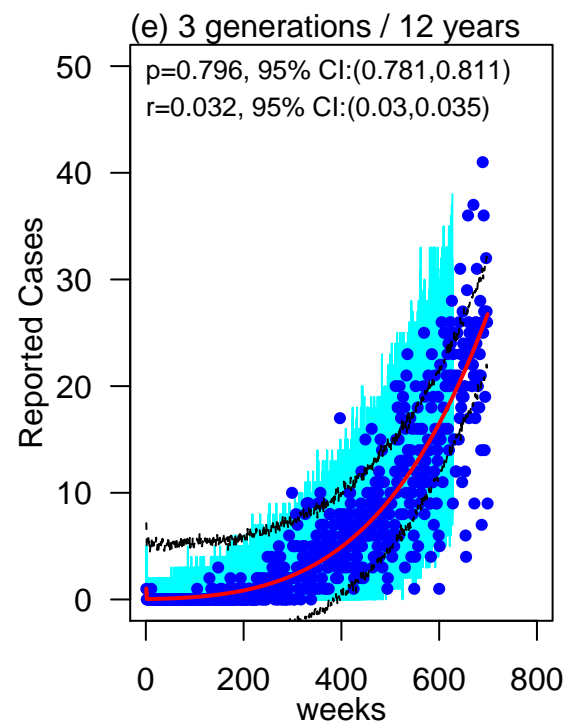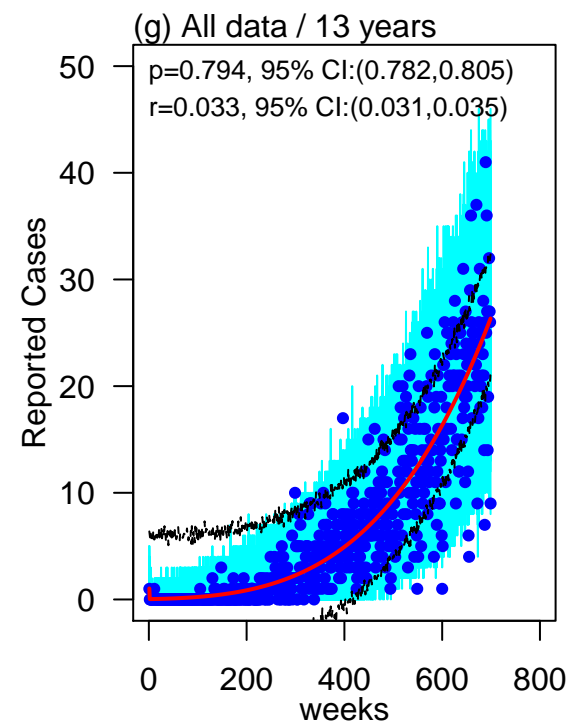

Supplement: S4 Fig — We fit generalized-growth model during various epidemic phases for MSM reported HIV cases in Shenzhen and estimated the corresponding parameters r, p and their 95% CIs. In panels (a) and (b), red solid lines indicate the parameter estimates of r and p to various epidemic generations. The shaded region and blue-dashed line indicates their corresponding 95% CIs with respect to various epidemic generations. Panels (c) to (g) present the fitting results of the generalized-growth model to epidemic generations 1 to 4, and all data, with a 95% prediction interval. Red solid lines indicate the simulation result. Blue dots indicate the weekly reported cases. Cyan lines indicate the sample of 1,000 Poisson simulations. (PDF) [file pone.0192849.s004.pdf]
